# Supplementary material for: Hidden in plain sight: what remains to be discovered in the eukaryotic proteome?
Source: Open Biol. 2019 Feb 20;9(2):180241. doi: 10.1098/rsob.180241 (PMC6395881; doi:10.1098/rsob.180241)
Supplement: Figure S1 [file rsob180241supp1.pdf]

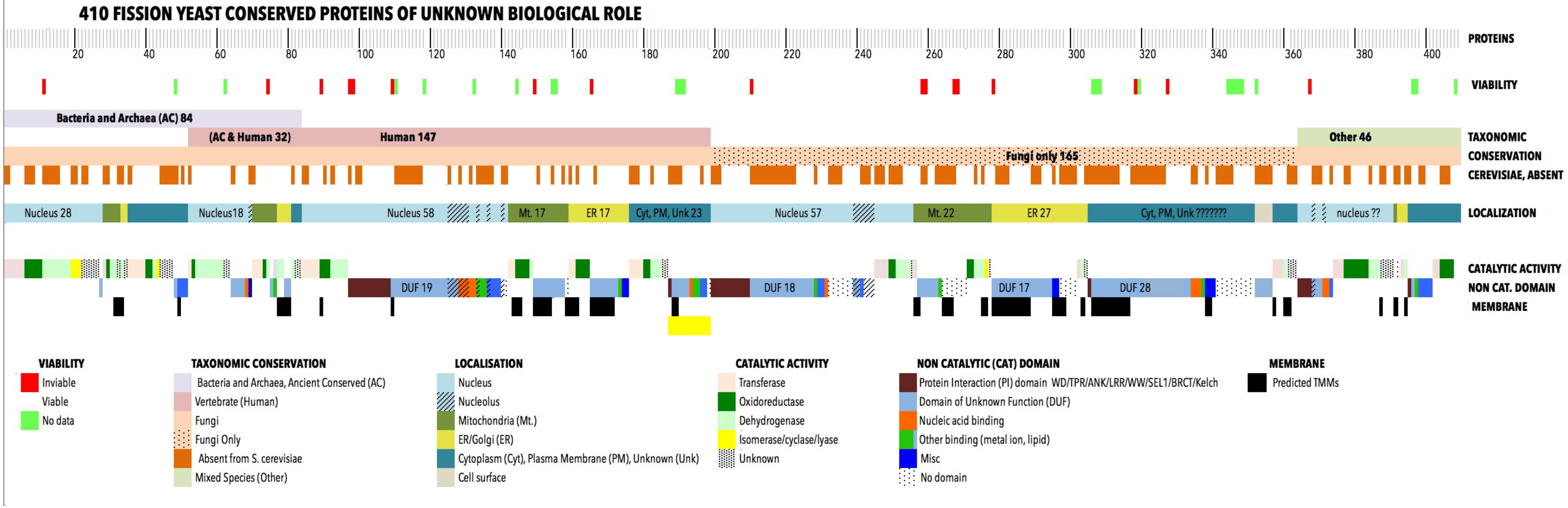

410 FISSION YEAST CONSERVED PROTEINS OF UNKNOWN BIOLOGICAL ROLE
20
40
60
80
100
120
140
160
180
200
220
240
260
280
300
320
340
360
380
400
PROTEINS
VIABILITY
Bacteria and Archaea (AC) 84
(AC & Human 32)
Human 147
Other 46
Fungi only 165
TAXONOMIC
CONSERVATION
CEREVISIAE, ABSENT
Nucleus 28
Nucleus18
Nucleus 58
Mt. 17
ER 17
Cyt, PM, Unk 23
Nucleus 57
Mt. 22
ER 27
Cyt, PM, Unk ???????
nucleus ??
LOCALIZATION
DUF 19
DUF 18
DUF 17
DUF 28
CATALYTIC ACTIVITY
NON CAT. DOMAIN
MEMBRANE
VIABILITY
Inviable
Viable
No data
TAXONOMIC CONSERVATION
Bacteria and Archaea, Ancient Conserved (AC)
Vertebrate (Human)
Fungi
Fungi Only
Absent from S. cerevisiae
Mixed Species (Other)
LOCALISATION
Nucleus
Nucleolus
Mitochondria (Mt.)
ER/Golgi (ER)
Cytoplasm (Cyt), Plasma Membrane (PM), Unknown (Unk)
Cell surface
CATALYTIC ACTIVITY
Transferase
Oxidoreductase
Dehydrogenase
Isomerase/cyclase/lyase
Unknown
NON CATALYTIC (CAT) DOMAIN
Protein Interaction (PI) domain WD/TPR/ANK/LRR/WW/SEL1/BRCT/Kelch
Domain of Unknown Function (DUF)
Nucleic acid binding
Other binding (metal ion, lipid)
Misc
No domain
MEMBRANE
Predicted TMMs
